# A Case Report on Transient Erythroblastopenia of Childhood in a Female Pediatric Patient

**DOI:** 10.7759/cureus.26451

**Published:** 2022-06-30

**Authors:** Umberto M Donato, Andrew Galligan

**Affiliations:** 1 Pediatric Oncology, Tampa General Hospital, Tampa, USA; 2 Radiology, Moffitt Cancer Center, Tampa, USA; 3 Pediatric Oncology, USF Health, Tampa, USA; 4 Pediatric Oncology, Moffitt Cancer Center, Tampa, USA; 5 Pediatric Hematology Oncology, University of South Florida Morsani College of Medicine, Tampa, USA

**Keywords:** anemia, pediatrics, prbc, red cell aplasia, hematology, reticulocytosis, transient erythroblastopenia of childhood

## Abstract

Transient erythroblastopenia of childhood (TEC) is an uncommon, benign normocytic anemia of unknown etiology. It is characterized by absent or reduced erythroid precursors in otherwise normocellular bone marrow, with patients undergoing a complete spontaneous recovery. We present the case of a 12-month-old female suspected of TEC.

## Introduction

Transient erythroblastopenia of childhood (TEC) is a slowly developing anemia, first identified in 1970 [1], that occurs mainly in early childhood and is distinguished by a stable onset of pallor. Patients are usually between six months and six years old. As stated by the name of the disease, all patients diagnosed with TEC recover completely with unremarkable sequelae. Along these lines, patients with TEC are mostly healthy. The etiology of TEC is unestablished but researchers have found numerous pathways that involve viral and immunologic mechanisms [2]. More recently prospective analyses of similar cases have failed to localize a specific viral agent responsible for TEC [3]. However, they often present with typical anemic symptoms such as mucosal pallor, cardiac flow murmurs, and tachycardia [4]. Parents of the patients often mention reduced energy levels and elevated fatigue in children with this disease. Complete blood count (CBC), hemoglobin studies, reticulocyte count, and viral studies are indicated for patients suspected of TEC. Bone marrow studies may be considered as well when physical exam and CBC data are inconsistent with a TEC case. Red cell adenosine deaminase (RCAD) levels have also been used in the past to distinguish between Diamond-Blackfan anemia, TEC, and other anemias as well. In patients with TEC, RCAD levels are typically either depressed or normal. With regard to the management of the disease, packed red cell transfusions are the standard form of treatment in patients with severe presentations of TEC with altered mental status and hemodynamic instability.

TEC is an extremely rare pathology with a prevalence of 4.3 cases per 100,000 children [5]. Due to its rarity, a further understanding of the different laboratory and clinical features of this disease will allow physicians to avoid redundant interventions and procedures in order to provide a prompt diagnosis. We present the case of a 12-month-old female diagnosed with TEC.

## Case presentation

The patient is a previously healthy 12-month-old female with no significant medical history besides a prior emergency department admission for dehydration from viral gastroenteritis. History obtained from the father indicated she presented with “fussiness” and decreased oral intake for the past two weeks. Father stated the patient was reluctant to walk and would usually sit in one place all day. The day she was brought to our pediatric clinic, the patient had an episode of non-bilious/non-bloody emesis after attempting to eat early in the morning. The patient was unremarkable for fever, ear tugging, and cough but did have a runny nose that had not resolved for the past week. The patient was seen a week before by the primary care provider who felt the patient was suspicious for a viral upper respiratory tract infection. Since this last visit, the female has lost 700 grams and has looked increasingly pale. Family history was remarkable for a sickle cell trait diagnosis on the paternal side of the family, a cousin. Physical exam was notable for irritability but no lethargy. Mucosal and hand pallor were noticed. The exam was also remarkable for a pronounced cardiac flow murmur. As far as vitals, the patient was tachycardic, slightly hypertensive, and had an elevated respiratory rate (Table 1). Complete blood count results were notable for a normocytic, profound anemia (hemoglobin/hematocrit: 1.7/5.6 g/dL) with otherwise intact WBC and platelet counts (Table 2). Parvovirus, uric acid, and respiratory viral panel results were all unremarkable. Flow cytometry data revealed severe anemia with leukoblastosis. The remaining complete metabolic panel results were within reference values. Blood smear revealed no RBC abnormalities and was vastly unremarkable for any qualitative defects.

**Table 1:** Vital signs

	Values at presentation (Day 1)	Values at discharge (Day 3)	Reference Values
Temperature (C)	36.8	36.5	35.5-37.5
Pulse/min	153	96	80-140
Respirations/min	30	26	20-30
Blood pressure	108/51	100/60	80-110/53-66
Pulse Oximeter (%)	97	100	95-100

**Table 2:** Complete blood count values

	Values at presentation (Day 1)	Values at discharge (Day 3)	Reference Values
White blood cell (10*3/uL)	9.38	9.98	5.00-17.00
Red blood cell (10*6/uL)	0.86	2.68	3.80-5.40
Hemoglobin (g/dL)	2.3	7.5	10.5-13.5
Hematocrit (%)	7.0	22.4	33.0-40.0
Mean corpuscular volume (fL)	81.4	83.6	70-87
Mean corpuscular hemoglobin (pg)	26.7	28.0	27-31.2
Mean corpuscular hemoglobin concentration (g/dL)	32.9	33.5	30.0-37.0
Platelet count (10*3/uL)	437	465	142.0-424.0
Mean platelet volume (fL)	9.0	8.9	9.4-12.4
Red cell distribution width (%)	13.0	14.1	11.6-14.6
Nucleated red blood cells ( /100 RBCs)	0.0	0.2	0.0-0.2
Total nucleated red blood cells (10*3/uL)	0.00	0.02	0.00-0.02

Differential diagnoses included transient erythroblastopenia of childhood (TEC), congenital bone marrow failure, Diamond-Blackfan anemia (DBA), infectious disease given normocytic anemia, and reticulocytopenia (Table 3). However, such low hemoglobin (1.7) is not often found in TEC but is seen in DBA. Fanconi's anemia was less likely, given the absence of pancytopenia and congenital anomalies. Transferrin, iron, total iron-binding capacity, and ferritin were within standard values. Vitamin B12 and folic acid data were unremarkable and thus less concerning for a nutritional deficiency. Blood smear was unremarkable as stated earlier. RBCs were 7-8 um in diameter and anucleated in the smear. Slight elevation of LDH, normal haptoglobin, normal uric acid, negative Coombs, normal total bilirubin, no hepatosplenomegaly, and reticulocytopenia made it less concerning for hemolysis. WBC counts were normal as well making it less likely for a neoplastic malignancy. Given these values and the differentials regarding the anemic symptoms, the patient was started on slow packed-RBC (pRBC) transfusions of 40 mL/kg over two hours in order to avoid inducing heart failure. No red-cell adenosine deaminase values were recorded before transfusion.

**Table 3:** Other Hematological Values

Table 3: Other Hematological Values			
	Values at presentation (Day 1)	Values at discharge (Day 3)	Reference Values
Reticulocyte (%)	0.81	1.24	0.6-1.6
Immature reticulocyte fraction (%)	12.7	33.3	2.3-15.9
Absolute reticulocyte (10*6/uL)	0.0056	0.0332	0.03-0.09
Reticulated hemoglobin (pg)	42.8	35.10	30.0-38.0

After transfusion, she was sent to the pediatric intensive care unit for continued transfusions and hematologic monitoring. In total, the patient received three pRBC transfusions over the course of six hours. Hemoglobin/hematocrit after each transfusion revealed 2.3/7.0 → 4.4/13.0 → 7.1/21.2. Reticulocyte count rose to 1.24% within a few hours of the last transfusion. Consequently, the patient was discharged pending bone marrow biopsy results which were followed up to be normal (Table 3). Heart murmur also resolved post-transfusion as seen in a month of follow-up. Considering, the main signs/symptoms and lab findings of our patient at presentation, such as skin and mucosal pallor, tachycardia, a cardiac flow murmur, reticulocytopenia, and a spontaneously resolving normocytic anemia, our patient was diagnosed with TEC. Altogether, this particular case was mostly consistent with TEC presentations seen in the literature [4].

## Discussion

TEC falls under the larger category of pure red cell aplasias and it is usually defined as anemia with a hemoglobin level less than two standard deviations (1.0 g/dL) [6] under reference values [7]. TEC symptoms also include a reduced reticulocyte count alongside absent evidence for other causes of the anemia. There is scarce evidence of recurrent TEC cases and no interventions besides standard supportive measures. PRBC transfusions in severe cases are required to raise hemoglobin and red blood cell levels in patients. Median hemoglobin in TEC patients is reported to be close to 4.4 g/dL, absolute reticulocyte count 0 × 10(9)/L; nearly half of all patients are neutropenic and most have platelet counts of more than 400 × 10(9) /L [8]. These values were in concordance with our patient except for the hemoglobin and the neutropenia which our patient never experienced. By itself, TEC is quite rare [5], but to our knowledge, a TEC patient with hemoglobin values more than two standard deviations under the median for the disease has not been reported in the literature to date. Such a finding is worth documenting in order to prevent physicians from incorrectly diagnosing these patients based on hemoglobin reference values for the pathology.

One of the most important indicators of a TEC patient beginning recovery is the presence of reticulocytosis. The median interval for reticulocytosis in TEC is said to be near 12 days from symptom onset [9]. Our patient’s parents mentioned the child had experienced symptoms suspicious for anemia nearly 16-17 days before reticulocytosis was evidenced. It can thus be said our patient more likely experienced “late-onset” reticulocytosis due to the severity of her anemia by the time she came to the clinic (Table 3). Furthermore, this case was serologically negative for a parvovirus B19 infection which has been cited in the literature to be linked to TEC onset [10]. Altogether, the lack of a viral cause, late-onset reticulocytosis, absent neutropenia, and most notably, the greatly decreased hemoglobin values of our patient detail a unique presentation of TEC in a 12-month-old female. 

## Conclusions

Due to the varied presentations and rarity of TEC, it is important to identify this pathology early in order to avoid excessive interventions and misdiagnoses.
